# Phenotypic variation in hypocotyl elongation among elite sand rice (*Agriophyllum squarrosum*) lines

**DOI:** 10.1002/ece3.70051

**Published:** 2024-08-07

**Authors:** Yujie Liu, Xiaoyun Cui, Xiaofeng Li, Ruilan Ran, Guoxiong Chen, Pengshan Zhao

**Affiliations:** ^1^ Key Laboratory of Ecological Safety and Sustainable Development in Arid Lands Northwest Institute of Eco‐Environment and Resources, Chinese Academy of Sciences Lanzhou China; ^2^ Key Laboratory of Stress Physiology and Ecology in Cold and Arid Regions, Gansu Province Northwest Institute of Eco‐Environment and Resources, Chinese Academy of Sciences Lanzhou China; ^3^ University of Chinese Academy of Sciences Beijing China; ^4^ Academy of Plateau Science and Sustainability, Qinghai Normal University Xining China

**Keywords:** *Agriophyllum squarrosum*, climate factors, geographic pattern, hypocotyl elongation, Partial Least Squares Path Modeling (PLSPM), phenotypic variations, seedling emergence

## Abstract

Sand rice (*Agriophyllum squarrosum*), widely distributed in Central Arid Asia and prevalent in the sand dunes of northern China, presents a promising potential as a climate‐resilient crop. The plasticity of hypocotyl growth is the key trait for sand rice to cope with wind erosion and sand burial, ensure seedling emergence, and determine plant architecture. In this study, we assessed the overall hypocotyl phenotype of six sand rice elite lines, which were collected from different regions of northern China, and selected by our group over past decade through common garden trials. Significant phenotypic variations were observed in thousand‐seed weight (TSW), seedling emergence percentage, hypocotyl length and diameter, and seedling fresh weight among the lines. The elite line Aerxiang (AEX) exhibited excellent agronomic performance with superior and synchronous emergence, and high survival percentage, distinguishing itself as a prime candidate for further large‐scale cultivation. Contrastingly, the lines from the arid regions showed markedly lower performance. Partial Least Squares Path Modeling (PLSPM) was used to assess the impact of seed provenance climate factors, including annual mean temperature (AMT) and annual mean precipitation (AMP), on trait variability among lines. The findings indicate a significant correlation between climate factors and hypocotyl length, highlighting the intricate adaptation of sand rice to local climate. The comprehensive understanding of the mechanisms behind phenotypic variations offers valuable insights for sand rice de novo domestication and innovative germplasm resources, and lays the foundation for ecological restoration in sandy areas.

## Graphical abstract

Sand rice presents a promising potential crop due to its wide distribution, comprehensive nutritional profile, and adaptability to extreme arid desert. Since 2010, our group has dedicated extensive efforts to the domestication and breeding of sand rice, resulting in the selection of several elite lines from 75 natural populations. This study analyzed the hypocotyl traits of six elite lines, revealing significant differences both among and within the lines. Partial Least Squares Path Modeling (PLSPM) analysis indicated a significant correlation between climate factors and hypocotyl length, exposing the intricate adaptation of sand rice to local environment.

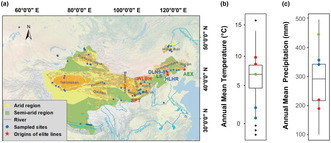

## 1 INTRODUCTION

Sand rice (*Agriophyllum squarrosum*), an annual psammophyte of the Amaranthaceae *sensu lato* family within the order of Caryophyllales, is extensively distributed in Central Arid Asia and thrives in arid and semi‐arid regions of northern China, notably on mobile sand dunes (Chen et al., 2014; Zhao et al., 2014). The nutritional profile, long‐term consumption history, high tolerance to environmental stresses, and ecological importance make sand rice a crop with significant potential for development (Chen et al., 2014; Zhao, Li, Sun, et al., 2023; Zhao, Ran, Li, et al., 2023; Zhao, Ran, Sun, et al., 2023). It can meet the demands for diversity in food sources, thereby enhancing food security (Chen et al., 2014; Xu, 2020; Zhao, Li, Sun, et al., 2023; Zhao, Ran, Li, et al., 2023). Since 2010, our group has dedicated extensive efforts to the domestication and breeding of sand rice (Chen et al., 2014; Zhao, Ran, Sun, et al., 2023). After a series of introduction, cultivation, and field selection breeding conducted at premier research facilities, notably the Shapotou Desert Research and Experiment Station and the Gaolan Station of Agricultural and Ecological Experiment, we have selected several lines with high adaptability and favorable agronomic traits from 75 natural populations (Zhao, Ran, Li, et al., 2023; Zhao, Ran, Sun, et al., 2023). Among them, Aerxiang (AEX) line exhibits stable phenotypic characteristics and possesses the best comprehensive agronomic traits, with a maximum seed weight per plant reaching 209 g, and is thus named after Professor Guoxiong Chen as GX‐1 (Zhao, Ran, Li, et al., 2023).

The growth and development of the hypocotyl undergo two critical stages: etiolation and photomorphogenesis. Photomorphogenesis is a complex process involving the actions of various photoreceptors, including phytochromes, cryptochromes, phototropins, and ultraviolet receptors such as UVR8, which regulate the transcription and expression of downstream genes to fine‐tune plant responses to changes in the external light environment (Xu et al., 2020). The hypocotyl length is a key growth factor for sand rice to overcome wind erosion and sand burial, ensuring seedling emergence on mobile sand dunes (Zhao, Ran, Li, et al., 2023; Zhao, Ran, Sun, et al., 2023). It has been showed that the hypocotyl length of sand rice varies significantly across regions with different levels of wind‐sand activity (Zhao, Ran, Sun, et al., 2023). The emergence percentage of sand rice seedlings exhibits a negative correlation with burial depth in sand (Cui et al., 2007; Tobe et al., 2005; Wang et al., 1998; Zheng et al., 2005). For example, the emergence percentage of sand rice seedlings from the Mu Us and Horqin sand fields decreased by 97.6% and 69.6%, respectively, as the burial depth in sand increased from 0.5 cm to 4 cm (Cui et al., 2007; Zheng et al., 2005). The plastic elongation of the hypocotyl significantly affects the seedling vigor and plant architecture, which is a core trait in the de novo domestication of sand rice (Zhao, Ran, Li, et al., 2023; Zhao, Ran, Sun, et al., 2023). Therefore, investigation and characterization of the overall hypocotyl phenotype is essential for identifying the most suitable sand rice lines with desired agronomic traits and strong adaptability for successful domestication and cultivation. In this study, we conducted a preliminary analysis on the phenotypic variation of multiple traits related to the hypocotyl in six elite lines developed from the natural populations. Traits examined included thousand‐seed weight (TSW), hypocotyl length and diameter, and seedling fresh weight, as well as the influence of climate factors. Our research contributes to the development of compact plant architecture and lodging‐resistant cultivars, paving the way for the de novo domestication of sand rice. This provides important plant resources for ecological restoration and the supply of diverse forage and pseudocereal in the sandy and windy areas of northern China.

## 2 MATERIALS AND METHODS

### 2.1 Plant materials

**FIGURE 1:**
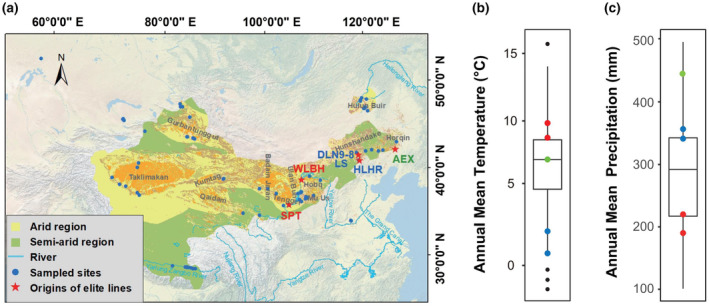
Geographical distribution and local climatic conditions of sand rice sampling sites and origins of elite lines. (a) Map illustrating the distribution of sand rice in Central Arid Asia and northern China. Blue circles indicate locations where sand rice seeds were sampled from natural populations, while red pentagons denote the origins of the six elite lines. The background imagery is from GEBCO_2014 Grid, version 20150318 (http://www.gebco.net), and the desert dataset (in orange) is provided by Geographic remote sensing ecological network platform (www.gisrs.cn). The arid regions and semi‐arid regions of China are highlighted in yellow and green, respectively, with data sourced from Natural Earth (https://www.naturalearthdata.com/). (b, c) Climatic conditions at sand rice distribution sites. Red circles represent the elite lines (WLBH, SPT) found in arid regions, blue circles represent the elite lines (HLHR, DLN9‐8, LS) in semi‐arid regions, and green circle represents the AEX elite line in semi‐humid region. Climate data were extracted from WorldClim (https://www.worldclim.org/version2) based on the geographic information of each sampling site using the R script as previous description (Zhao et al., 2022). Information on the deserts/sand fields was obtained from previous works (Zhao et al., 2022; Zhao, Ran, Li, et al., 2023).

Between 2010 and 2023, a total of 75 natural populations of seeds were sampled across major deserts and sand fields in China and Kazakhstan (detailed information available in the datasheet; Appendix S1). For each population, the seeds were harvested from independent individuals at least 30 m apart between September and the following February. The seeds were stored in the refrigerator at −20°C. After common garden trial, six elite lines selected by our group from natural populations are derived from arid regions of the Tengger desert (Shapoutou, SPT) and the Ulan Buh desert (Wulanbuhe, WLBH), semi‐arid regions of the Hunshandake sand field (Duolunnan9‐8, DLN9‐8; Longspike, LS; and Haolaihure, HLHR), and a semi‐humid region of the Horqin sand field (Aerxiang, AEX), respectively (Figure 1a). The geographic coverage of these elite lines spans latitudes from 37.47 to 42.87° N and longitudes from 105.02 to 122.43° E, with an altitude variation between 254 and 1351 m. These regions experience annual mean precipitation (AMP) between 191 and 490 mm and annual mean temperature (AMT) ranging from 1.13°C to 9.28°C (Figure 1a–c). In 2021, the six elite line seeds were planted and harvested at the Gaolan station and then stored for 15 months at 4°C in the laboratory from November 2021 to February 2023.

### 2.2 Growth conditions

Three individuals from WLBH and five individuals from each of five other elite lines (AEX, SPT, HLHR, DLN9‐8, and LS) were chosen for the experiments. Sixty‐five plum seeds per individual were sown in pots (7 × 7 × 8 cm), each containing 13 seeds and buried with 1 cm of sand from the Shapotou Desert Research and Experiment Station, Tengger desert. A total of 25 pots per elite line except for 15 pots of WLBH were placed on a tray, which was then positioned on a light stand set up in the laboratory. The top of the pots was approximately 25 cm away from a full‐spectrum plant growth supplemental light (three 15 W mixed white light tubes and two 30 W mixed red and blue light tubes), providing a photon flux density (PPFD) of 220 μmol m^−2^ s^−1^ (Figure S1), as measured by a photometer with a quantum sensor (SPIC‐300, EVERFINE Corporation, China). The plants were kept under a 14/10 h light/dark daily cycle with a maintained temperature of approximately 25°C and a relative humidity of 22.5%. To ensure proper emergence, the pots were watered once with 200–250 mL of distilled water at 9:00 a.m. every day to keep the sand surface moist with a humidity level of around 14%. After emergence, the pots were watered once with 200–250 mL of distilled water every 2 days until no further seedlings appeared. Trays were rotated twice daily to minimize position effects.

### 2.3 Measurements

For the measurement, five individuals from each elite line, except for WLBH which had three individuals, were selected. The thousand‐seed weight (TSW) was obtained by weighing and averaging five replicates of 50 randomly selected seeds from each individual, amounting to a total of 250 seeds per individual. Fifty seeds per individual were randomly selected to measure the major axis length using the ImageJ software. After seedling emergence, data collection was performed when the first pair of cotyledons in the sand rice seedlings had fully expanded and the first pair of true leaves had reached approximately 2 mm in length. The aerial parts of the seedlings were clipped and weighed to obtain the seedling weight, and photographs were immediately taken to measure the hypocotyl length and diameter using ImageJ. Meanwhile, the number of seedling emergence was recorded daily to determine the emergence indices for each elite line.

### 2.4 Statistical analysis

Normality tests using the Kolmogorov–Smirnov test with SPSS 16.0 (SPSS Inc., Cary, NC, USA) were performed on the parameters including emergence percentage, seedling weight, and hypocotyl length and diameter for each seedling. One‐way ANOVA followed by Kruskal–Wallis tests were used to compare genotypes and determine significant differences between them (*p* < .05). Partial least squares path (PLS‐PM) was utilized to explore the direct, indirect, and interactive relationships between climate factors and all measured variables using the R package “plspm” (https://github.com/gastonstat/plspm). The model incorporated the following variables: climatic factors (annual mean temperature, AMT, and annual mean precipitation, AMP), seed factors (seed major axis length and TSW), seedling weight, and hypocotyl length. Indirect effects were defined as multiplied path coefficients between predictor and response variables including all possible paths except for the direct effect. The optimal model among all constructed models was selected based on the goodness of fit (GoF) statistic, an indicator of the model's overall predictive capability. All analytical results and graphical representations were generated using R software 4.3.1, ensuring accuracy in analysis and clarity in visualization.

## 3 RESULTS

### 3.1 Seedling emergence characteristics vary significantly among sand rice elite lines

**FIGURE 2:**
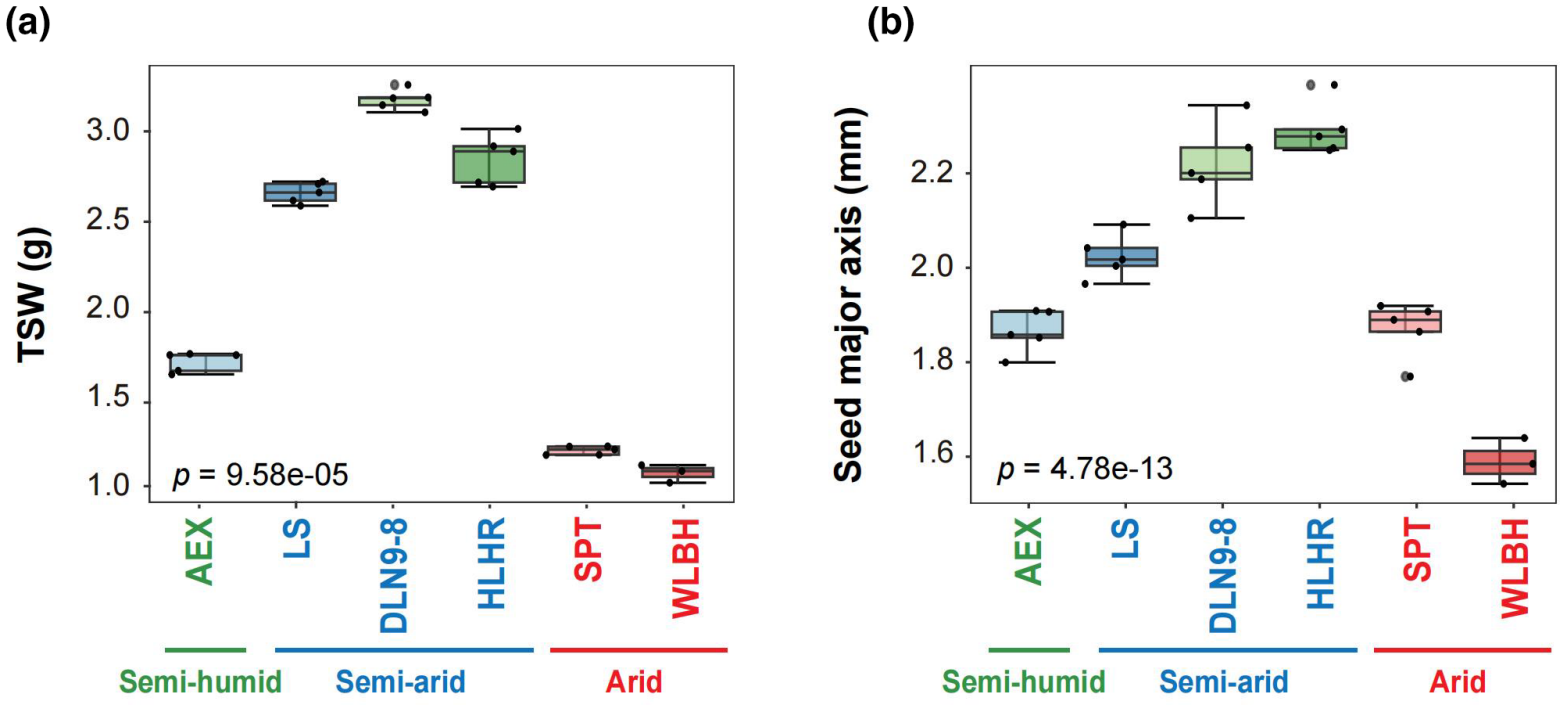
Seed mass across different geographical origins. (a) Thousand‐seed weight in six elite lines. Five individuals per line were measured, with five replicates of 50 seeds each. For WLBH line, only three individuals were included. The data represent the average of five replicates for each individual. (b) Seed major axis length was determined as the average measurement from 50 seeds per individual. Error bars indicate mean ± SD. One elite line (AEX) derived from a semi‐humid region is indicated in green, three elite lines (LS, DLN9‐8, and HLHR) from semi‐arid regions in blue, and two elite lines (SPT and WLBH) from arid regions in red.

**FIGURE 3:**
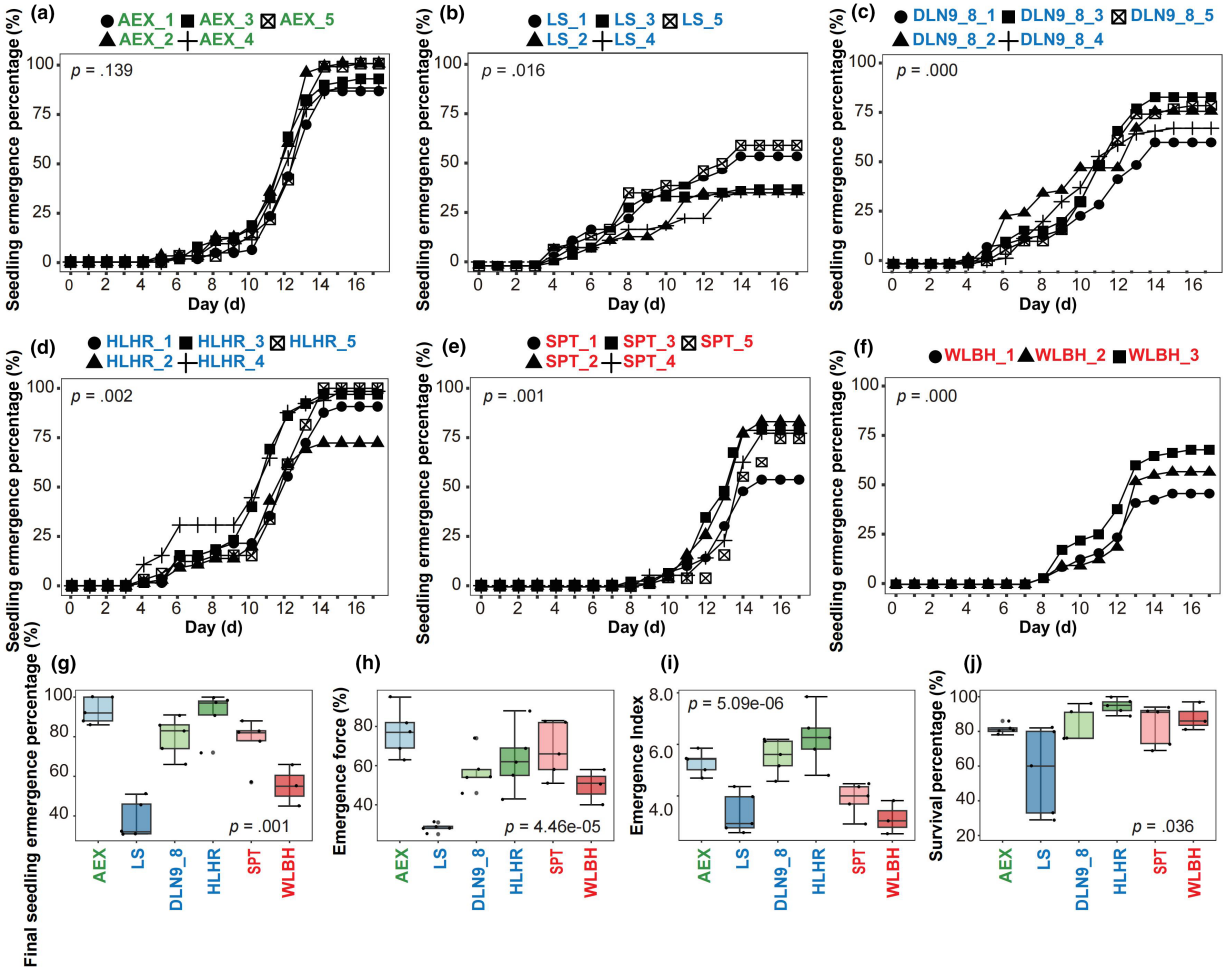
Phenotypic analyses of seedling emergence and survival for sand rice elite lines from different regions. (a–f) Seedling emergence percentages for elite lines from the semi‐humid region, semi‐arid regions, and the arid regions are indicated in green, blue, and red, respectively, as shown in Figure 2. Five lines (a–e) utilized seeds from five individual plants, with 65 seeds per plant, totaling 325 seeds per line for the experiments. Specifically, the WLBH line used seeds from three individual plants, totaling 195 seeds per line. (g) Final seedling emergence percentage of the six elite lines. (h, i) Emergence force and emergence index of the six elite lines. Emergence force = (Number of seedlings emerged up to the peak day/Total number of seeds tested) × 100%; the peak day is defined as the day with the maximum number of daily seedling emergence. Emergence index = ∑(Gt/Dt), in the formula: Gt is the number of emergences on day *t*, and Dt is the corresponding emergence days. (j) Survival percentages of the six elite lines.

The seed size and thousand‐seed weight (TSW) of the six elite lines showed significant variations, with average seed size ranging from 1.58 to 2.25 mm and TSW from 1.09 to 3.07 g (Figure 2a,b and Figure S2). Among the six elite lines, a significant difference in seedling emergence was observed (Figure 3a–g). The AEX line exhibited the highest final seedling emergence percentage, reaching 93.23% (ranging from 86.15% to 100%) (Figure 3a,g). The HLHR line had an average emergence percentage of 91.69% (with a range from 72.31% to 100%) (Figure 3d,g). The DLN9‐8 and SPT lines displayed intermediate seedling emergence percentage of 80.00% and 77.54%, respectively, within the ranges of 66.15%–90.77% for DLN9‐8 and 56.92%–87.69% for SPT (Figure 3c,e,g). The WLBH and LS lines had lower seedling emergence percentages, at 55.38% (ranging from 44.62% to 66.15%) and 38.15% (ranging from 30.77% to 50.77%), respectively (Figure 3b,f,g).

The elite lines originating from the Hunshandake sand field, including DLN9‐8, LS, and HLHR, were among the earliest to emergence, beginning on the fourth to fifth day post‐sowing and reaching peak daily emergence within 8–13 days. Their emergence forces were calculated at 46.15%–73.85%, 24.62%–30.77%, and 43.08%–87.69%, respectively. The emergence indices were 4.57–6.20, 2.58–4.36, and 4.80–7.85, respectively (Figure 3b–d,h). In contrast, the AEX line from the Horqin sand field initiated emergence on the fifth to sixth day post‐sowing. Daily emergence peaked on days 12–13, with an emergence force ranging from 63.08% to 95.38%. The emergence index was 4.69 to 5.85 (Figure 3a,h,i). The lines from the Tengger desert and Ulan Buh desert, SPT and WLBH respectively, began emergence later, starting on the eighth to ninth days post‐sowing, and reached peak daily emergence after 13 or 16 days, with emergence force ranging from 50.77% to 83.08% for SPT and 40.00% to 58.46% for WLBH. Their emergence indices were calculated at 2.91–4.47 for SPT and 2.53–3.82 for WLBH (Figure 2e,f,h,i). An analysis of intra‐line variability in emergence percentage revealed no significant differences within the AEX line (*p* = .139), while notable individual variations were found within the other five lines (*p* < .05), indicating a synchronous emergence pattern in the AEX line (Figure 3a–f). Survival percentages among lines were also compared. The HLHR line had the highest average percentage (94.63%), followed by WLBH, SPT, AEX, and DLN9‐8 lines (88.02%, 83.84%, 81.47%, and 72.01%, respectively). The LS line exhibited the lowest survival percentage at 56.74% (Figure 3j).

Comparative analysis of comprehensive traits during the emergence phase among lines indicated that the AEX line, originating from the eastern semi‐humid area, showed superior performance with high emergence percentage, synchronous emergence, and high survival percentage.

### 3.2 Phenotypic variations in hypocotyl traits across six elite lines

**FIGURE 4:**
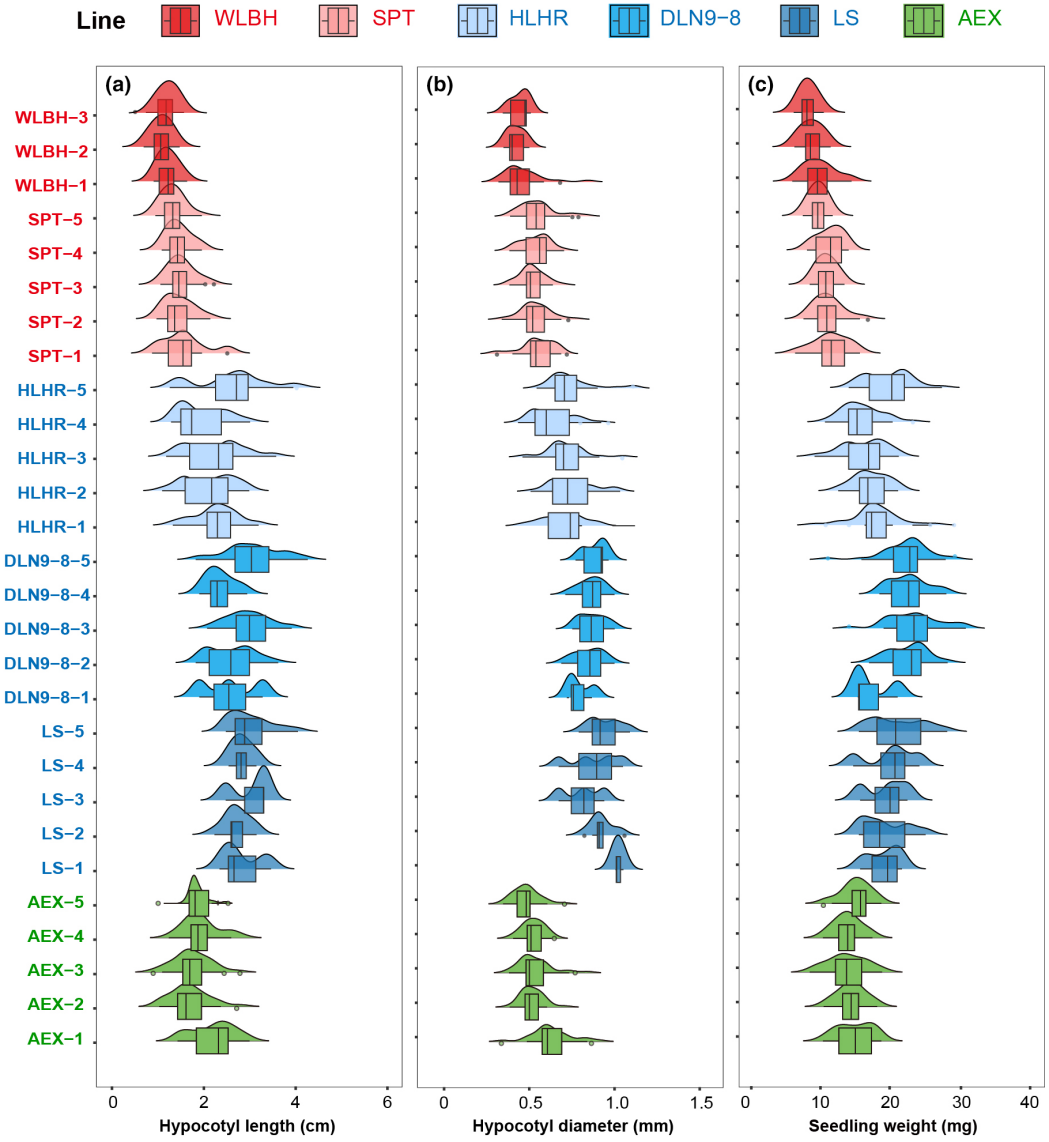
Comparative analyses of seedling phenotypes in six elite lines. (a–c) Statistical analysis of (a) hypocotyl length, (b) hypocotyl diameter, and (c) seedling weight for the six elite lines. Individual names of each line listed on the left are colored as those in Figure 2.

Comparative analyses of the hypocotyl length and diameter, and fresh weight of the aerial parts of seedlings among six elite lines revealed substantial phenotypic variation (Figure 4a–c and Figure S3). Hypocotyl lengths of the sand rice lines derived from a semi‐humid region (AEX) and semi‐arid eastern sand regions (DLN9‐8, LS, and HLHR) were significantly longer than those of lines from the arid regions (SPT and WLBH). Among them, the HLHR and DLN9‐8 lines had the longest hypocotyls, averaging 2.24 cm and 2.21 cm, respectively. LS and AEX lines followed, with lengths of 1.85 cm and 1.69 cm, respectively. In contrast, the SPT and WLBH lines had shorter hypocotyls, measuring 1.25 cm and 0.93 cm, respectively (Figure 4a). There was also a considerable within‐individual variation in hypocotyl length for each elite line; for instance, the HLHR line ranged from the shortest at 1.09 cm to the longest at 4.15 cm, the DLN9‐8 line ranged from 1.25 cm to 5.28 cm, the LS line ranged from 1.19 cm to 3.01 cm, the AEX line ranged from 0.69 cm to 3.28 cm, the SPT line ranged from 0.66 cm to 2.32 cm, and the WLBH line ranged from 0.29 cm to 1.43 cm (Figure 4a). The hypocotyl diameter and the fresh seedling weight of the aerial part also exhibited the similar pattern, with significant differences observed both between different lines and within each of the six lines (Figure 4b,c).

### 3.3 Effects of seed mass on hypocotyl length in sand rice

**FIGURE 5:**
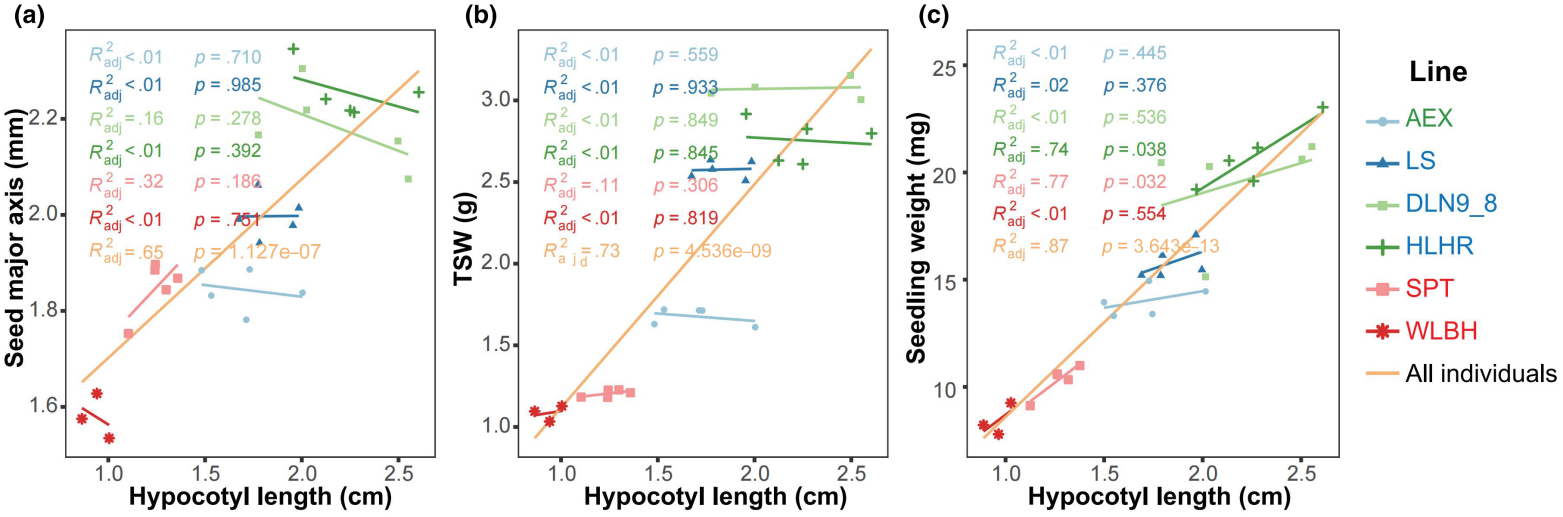
Regression analyses of hypocotyl length with different characteristics in sand rice elite lines. (a) Regression with seed major axis length, (b) TSW, and (c) seedling weight. The regression analyses within each elite line are shown in different colors as in legend description, whereas the orange line represents across all the individuals from six elite lines. *p* < .05 means a significant correlation.

To investigate the effects of seed mass on hypocotyl growth in sand rice, we analyzed the relationships between seed size, TSW, seedling fresh weight, and hypocotyl length. The results showed a highly significant correlation between these three parameters and hypocotyl length when all individuals from six elite lines were included ($R_{\mathrm{adj}}^{2}$ > .60, *p* < .01, Figure 5a–c). However, no correlation was detected between seed size or TSW and hypocotyl length within each of the six elite lines, except for a significant correlation between seedling fresh weight and hypocotyl length in the HLHR and SPT lines ($R_{\mathrm{adj}}^{2}$ > .70, *p* < .05). These findings suggest a complex interplay between seed size and hypocotyl length both among and within sand rice elite lines.

### 3.4 Climate influencing factors on seed mass and seedling characteristics of six elite lines

**FIGURE 6:**
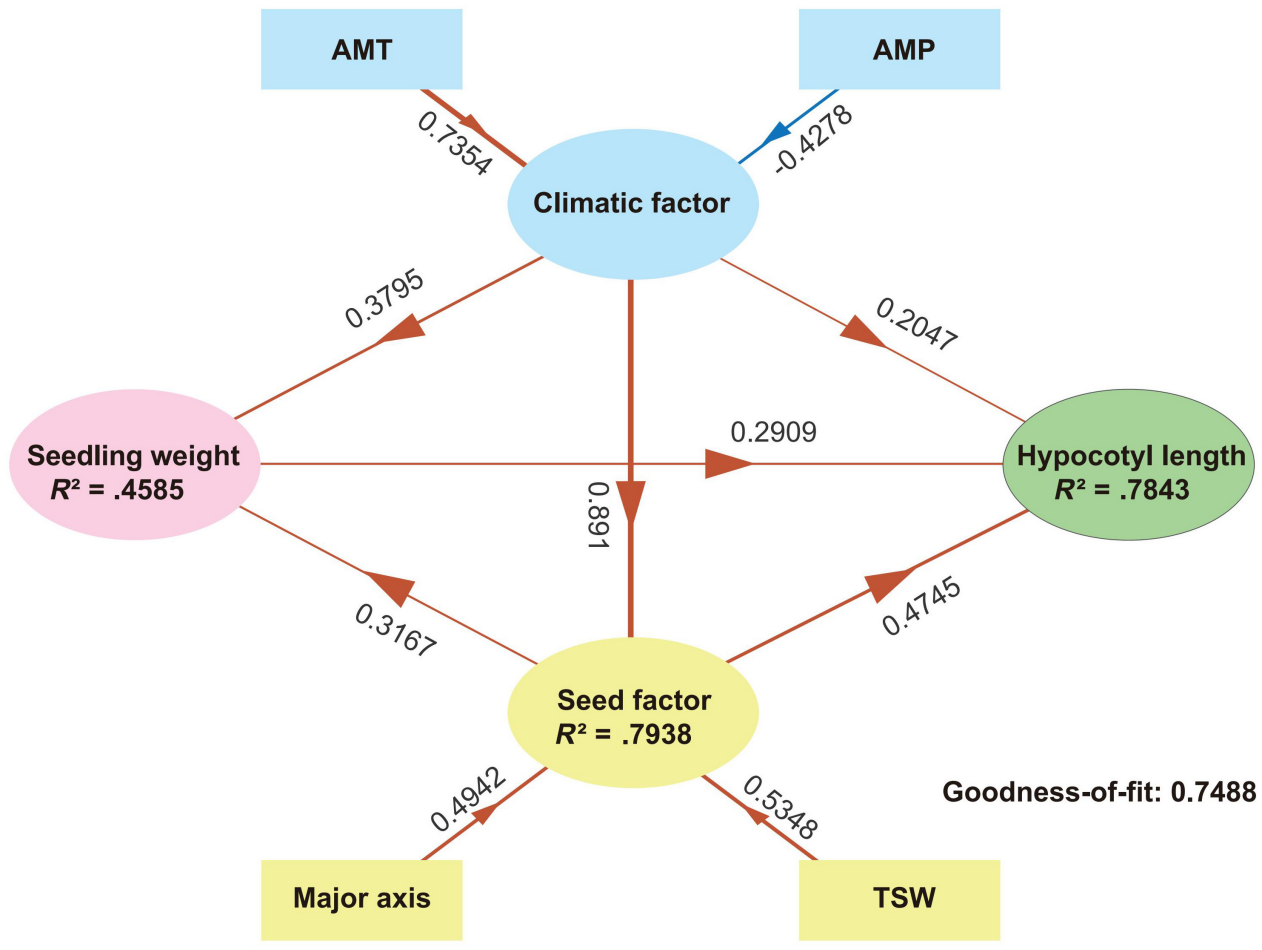
Partial least squares path modeling (PLS‐PM) showing the direct and indirect effects of different factors on seed and seedling characteristics. Observed variables are represented as rectangles, while latent variables are depicted as ellipses. Arrows from rectangles to ellipses indicate observed variable weights on latent variables, and arrows between ellipses represent the total effects among latent variables. Path coefficients are provided to elucidate the strength and direction of these effects. Orange and blue lines indicate positive and negative significant relationships, respectively. *R* ^*2*^ indicates the total variation of a dependent variable is explained by independent variables; the goodness of fit (GoF) of the entire model was .75.

A Partial Least Squares Structural Equation Modeling (PLS‐PM) was used to dissect the effects of climate factors on seed and seedling characteristics in six elite lines (Figure 6). The model showed that the latent variable climatic factor had a higher weight for AMT (0.74) than for AMP (−0.43), suggesting a more significant influence of AMT on the climatic factor. The climatic factor had a direct effect on hypocotyl length with a path coefficient of 0.20, indicating a moderate influence. Additionally, the climatic factor exerted significant impacts on both seed factor and seedling weight, with path coefficients of 0.89 and 0.38, respectively. Within the seed factor, both seed major axis length (0.49) and TSW (0.54) appeared to have a comparable influence. Moreover, the seed factor showed a notable positive effect on hypocotyl length with a path coefficient of 0.48. This finding emphasizes the seed factor as a pivotal mediator in the relationship between climatic conditions and hypocotyl length. In summary, these results reveal the predominant influence of AMT among climatic variables affecting hypocotyl length, which occurs directly, as well as indirectly through its effects on the seed factor and seedling weight. The positive contributions of the seed factor and seedling weight to hypocotyl length further highlight their crucial roles in the growth and development of sand rice.

## 4 DISCUSSION

Sand rice from different origins exhibit varying degrees of seed dormancy, presenting a continuous germination pattern during the growing season (Cui et al., 2007; Tobe et al., 2005; Wang et al., 1998; Zheng et al., 2005). In this study, it showed significant variations in seed size and TSW among different sand rice elite lines from different regions, as well as in seedling emergence percentage and synchronicity (Figure 2 and Figures S2 and S3). In one hand, this is likely due to differing nutrient accumulation causing by distinct climatic conditions (such as light, temperature, and precipitation) during seed development (Yin, Qian, et al., 2016; Yin, Zhao, et al., 2016; Zhao et al., 2022). On the other hand, the genetic composition of each elite line plays a fundamental role in their growth and developmental patterns, further contributing to the phenotypic differences in key traits, including seed size, weight, and emergence percentage (Liu et al., 2022; Nie et al., 2020; Qian et al., 2016; Qian et al., 2021; Yin, Zhao, et al., 2016; Zhao et al., 2017). Nonsynchronous emergence and weak seedling vigor could negatively affect the crop yield and quality, therefore, ensuring rapid and synchronous seedling emergence is important for the domestication and breeding of sand rice (Zhao, Ran, Li, et al., 2023). Notably, the AEX line exhibits a higher emergence percentage, enhanced synchronicity in emergence, and improved survival rate, indicating its superior performance during the domestication process. Hence, selecting elite lines with better emergence performance through artificial selection can enhance the adaptability and resilience of sand rice, thereby increasing its potential for survival and reproduction in extreme environments.

Significant differences in hypocotyl length were observed among sand rice elite lines (Figure 4 and Figure S3). This phenomenon could be attributed to multiple factors. Firstly, parental effects may have contributed to this variation (Zhao, Ran, Sun, et al., 2023), as complex correlations were found between seed size and hypocotyl length (Figure 5). Meanwhile, the plastic growth of sand rice hypocotyl can be influenced by environmental elements including temperature and humidity. Results from the PLS‐SEM model suggest annual mean temperature can directly impact hypocotyl length and also indirectly mediate the hypocotyl growth by affecting TSW and seedling fresh weight. Therefore, the hypocotyl length variation among different lines may imply that local adaptation has existed to cope with sand burial due to the various windy strength during seedling emergence in natural populations of sand rice. Interestingly, the model indicates that a decrease in precipitation is associated with an increase in the climatic factor, leading to an increase in seed mass, which contrasts with the findings by Zhao et al. (2022). Such discrepancy may stem from small number of elite lines included, plastic response to the environmental divergences between provenances and the breeding stations, as well as differences in analytical approaches. Furthermore, significant within‐individual variation in hypocotyl length was found for each of the six lines. These results suggest that phenotypic instability, rather than robustness, is heritably evolved in sand rice to buffer the heterogeneity of sand burial on windward slope, top, leeward slope, and foot of the same sand dune and the intermound lowland (Liu et al., 2024).

The variation in hypocotyl length and its plastic growth are very essential for sand rice to survive on shifting sand dunes. However, these weed‐like traits hamper us to develop it as a new crop, since the elongated hypocotyl is easily resulting in a lodging architecture, which in turn affects the large‐scale cultivation and reduces the yield. Therefore, selecting and breeding lines with compact plant architecture and lodging resistance is imperative for sand rice domestication and utilization (Zhao, Ran, Li, et al., 2023; Zhao, Ran, Sun, et al., 2023). Future efforts should be directed to optimizing sand rice hypocotyl length via selective breeding to cope with specific environmental stresses, such as wind and sand burial. By considering these factors in both research and application, we could better understand and exploit this plant genetic resource.

## AUTHOR CONTRIBUTIONS

**Yujie Liu:** Formal analysis (equal); investigation (equal). **Xiaoyun Cui:** Writing – original draft (equal); writing – review and editing (equal). **Xiaofeng Li:** Investigation (supporting). **Ruilan Ran:** Investigation (supporting). **Guoxiong Chen:** Conceptualization (supporting); writing – review and editing (supporting). **Pengshan Zhao:** Conceptualization (equal); writing – review and editing (equal).

## CONFLICT OF INTEREST STATEMENT

The authors declare that they have no competing interests.

## Supporting information


Figures S1–S3

Appendix S1

## Data Availability

All data supporting the findings of this study are available in the main text and in Figures S1–S3 and Appendix S1 provided online.
