# Supplementary material for: Phenotypic variation in hypocotyl elongation among elite sand rice (Agriophyllum squarrosum) lines
Source: Ecol Evol. 2024 Aug 7;14(8):e70051. doi: 10.1002/ece3.70051 (PMC11303975; doi:10.1002/ece3.70051)
Supplement: Supplementary file 1 — Figures S1–S3 [file ECE3-14-e70051-s002.docx]

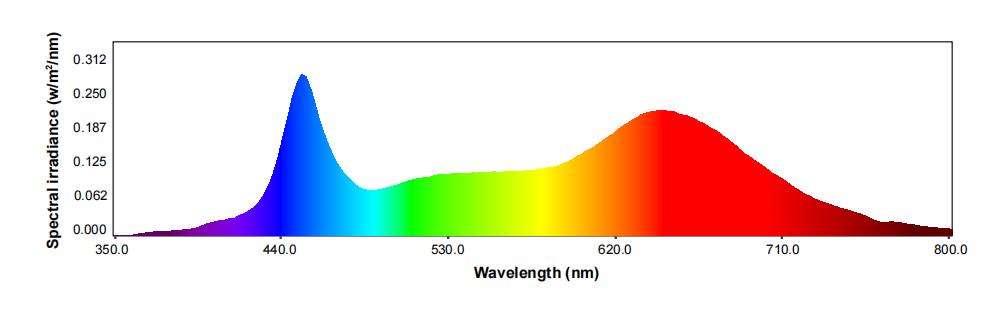


**Figure S1** Spectral irradiance settings for the experiment on the hypocotyl growth in the elite lines.


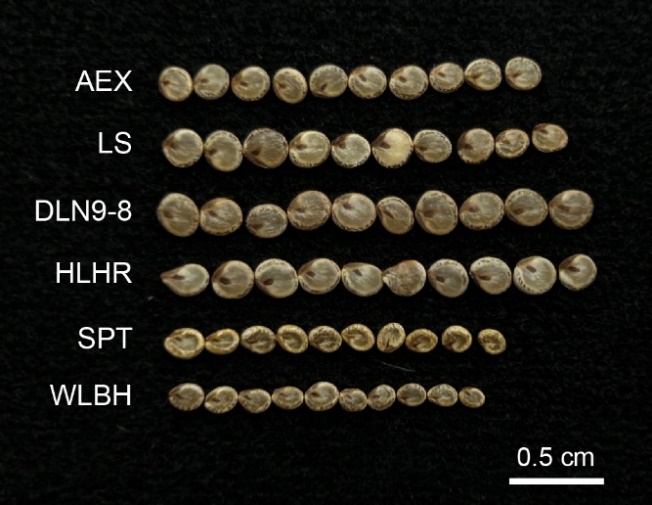


**Figure S2** Seed size phenotypes of six elite lines. Each line shows 10 seeds. Scale bar = 0.5 cm.


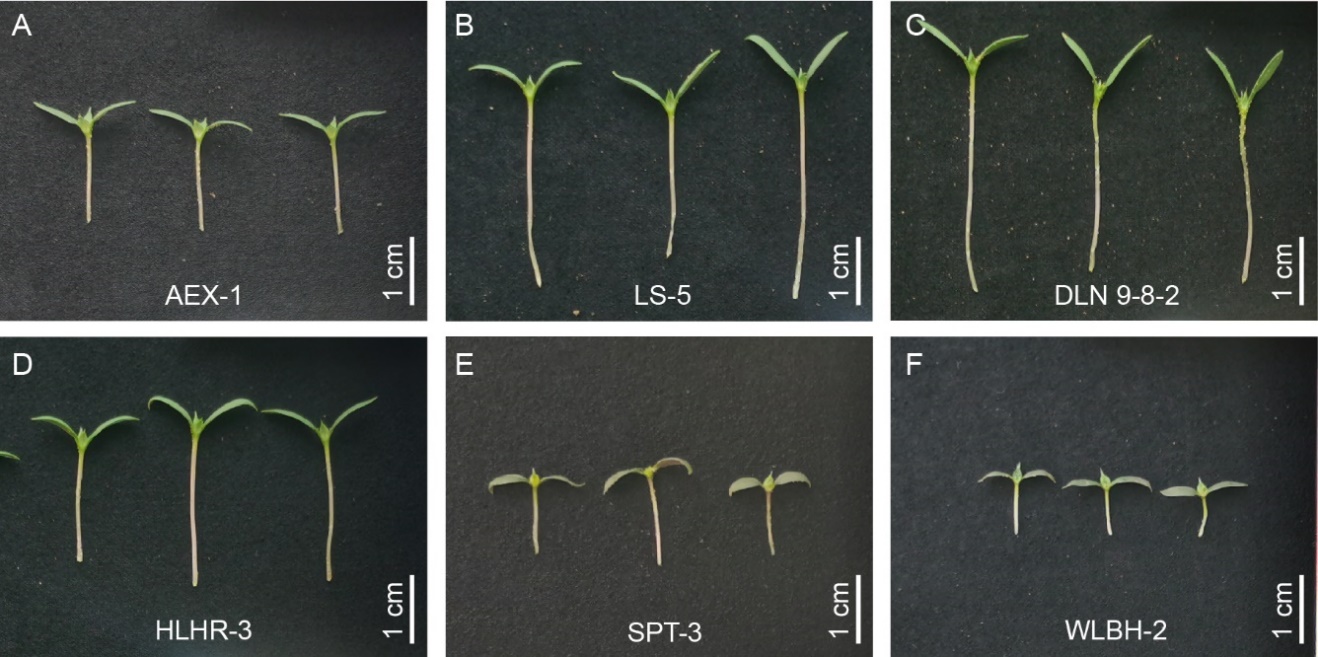


**Figure S3** Hypocotyl length variations among and within six sand rice elite lines. The aerial parts of the elite lines were photographed on February 19, 2023. Scale bar = 1 cm. Scale bar = 1 cm.
